# Diffuse Esophageal Spasm: An Alternative Treatment Approach

**DOI:** 10.7759/cureus.59822

**Published:** 2024-05-07

**Authors:** McKenzie K Allen, Wayne Frei

**Affiliations:** 1 Gastroenterology, Edward Via College of Osteopathic Medicine, Aiken, USA; 2 General Surgery, Aiken Regional Medical Center, Aiken, USA

**Keywords:** esophageal motility, esophageal motility disorders, distal esophageal spasm, corkscrew esophagus, diffuse esophageal spasm (des)

## Abstract

Diffuse esophageal spasm (DES) is a rare esophageal motility disorder characterized by abnormal contractions of the esophagus, leading to curling of the esophagus. The classic finding on barium swallow resembles that of the turns of a corkscrew. This case report presents a case of DES with impressive imaging and an alternative treatment approach.

There are no well-established guidelines for the treatment of DES. Treatment options include surgical myotomy, oral medications to aid in smooth muscle relaxation, esophageal dilation, and several newer approaches such as endoscopic botulinum toxin injections. There is a need for less invasive treatment modalities that provide a solution, longer than the duration of action of an oral medication in patients who are not candidates for surgical intervention. This case report presents a complex and challenging case of DES in the context of multiple complicating comorbidities. This case is unique in demonstrating the successful management of a rare esophageal motility disorder in a high-risk patient using a more traditional, less invasive treatment approach.

This case report presents a 91-year-old cachectic female with DES in the context of various comorbidities. Given her age, comorbidities, and current status, she was not a candidate for surgical intervention. After attempts at pharmacological therapy, the patient’s dysphagia continued to worsen. Upper gastrointestinal endoscopy with pneumatic esophageal dilation was performed. The patient’s swallowing improved in the days following endoscopic dilation.

## Introduction

Diffuse esophageal spasm (DES) is a rare esophageal motility disorder that most commonly presents as progressive dysphagia [1]. The disorder is associated with uncoordinated or rapidly occurring contractions of the smooth muscle of the esophagus that disrupt the normal peristaltic mechanism of swallowing [1]. The contractions are simultaneous and alternate with normal peristalsis [2]. DES most commonly occurs in white, elderly, females [1]. A definitive etiology of DES is unknown. One theory indicates that uncoordinated contractions are a result of a disrupted balance between inhibitory and excitatory postganglionic pathways [1]. Additionally, other possible theories stem from the distal part of the esophagus showing muscular hypertrophy or hyperplasia in most cases of DES [1].

The gold standard for the diagnosis of DES includes esophageal manometry [3]. First-line treatment includes calcium channel blockers and nitrates [1]. The second line includes endoscopic procedures such as botulinum toxin injections and pneumatic dilation [1]. The most feared complication of esophageal dilation is esophageal perforation [4]. The risk of perforation is low, but it is important to note that in the setting of perforation, there is a mortality of 20% and there is an association with underlying malignancy [1,4,5]. Esophageal botulinum injections have been shown to be beneficial in improving dysphagia in spastic disorders, such as DES, and in individuals diagnosed with achalasia who are elderly or have multiple comorbidities [6,7]. Studies indicating the benefit of this approach are small and primarily retrospective [6,7]. These studies indicate there is resolution of dysphagia for an average of six months following the injections [6,7]. More evidence is needed to fully support the use of botulinum injections as a primary treatment approach for DES.

Surgical approaches are reserved for particularly complex or refractory cases [2]. Surgical approaches, such as the Heller myotomy (through thoracotomy or with thoracoscopic access), have previously yielded better outcomes than endoscopic procedures [2]. Cases of DES that were treated with surgical approaches such as long myotomy of the esophagus and gastric cardia with a complete fundic patch have demonstrated promising outcomes [8,9]. This approach has decreased the risk of postoperative gastroesophageal reflux disease [8]. In elderly patients with a comorbidity or operative risk, surgery is often contraindicated. Another, less invasive surgical approach that was originally designed for the treatment of achalasia, peroral endoscopic myotomy (POEM) was first performed in 2010 [10]. A randomized control trial comparing POEM to the gold standard surgical approach in achalasia, Heller myotomy, is ongoing [10].

DES is often associated with increased lower esophageal sphincter pressure, which has been noted as a predictor of good responses to pneumatic dilation [5,11]. Symptom resolution after pneumatic dilation, has been recorded at as long as 10 to 25 years [11]. Younger patients tend to respond less favorably to pneumatic dilation [5]. Most studies conclude the symptoms most often recur in time in 50% or more of patients [4,5,11].

This case presents a complex scenario of a 91-year-old female with DES and multiple comorbidities. Metoclopramide and nifedipine were both trialed in this patient, as medications are the first-line treatment of DES [1]. The dysphagia continued to worsen and was successfully treated with a more traditional approach, pneumatic dilation. This case presents the successful management of a rare disorder in a patient with multiple comorbidities adding to the complexity of the case.

## Case presentation

A 91-year-old female with a recent COVID-19 infection, secondary bacterial pneumonia, and Clostridium difficile colitis presented with progressive dysphagia to solids and loss of appetite. The patient is a smoker with congestive heart failure and chronic obstructive pulmonary disease. A physical exam revealed a cachectic and malnourished female with a BMI of 16 and hyperchloremic metabolic acidosis (Table 1). The patient was in distress when swallowing. The patient initially presented to the emergency department in septic shock with persistent diarrhea secondary to Clostridium difficile colitis. Her diarrhea resolved with a 10-day course of fidaxomicin. The patient continued to have worsening leukocytosis (Table 1) throughout her hospital course despite treatment of her various conditions with fidaxomicin, tigecycline, linezolid, and meropenem. Her sputum culture was positive for Klebsiella pneumoniae, indicating aspiration pneumonia likely secondary to swallowing difficulties. Treatment with meropenem was initiated after the results of the sensitivity analysis were reviewed. The top differential diagnoses initially considered for the underlying cause of the patient’s dysphagia included esophageal strictures secondary to a history of gastroesophageal reflux disease (GERD), esophageal cancer, esophagitis, and esophageal motility disorder. The differential diagnoses of DES include angina, achalasia, scleroderma, esophageal cancer, gastroesophageal reflux disease, esophageal diverticula, other esophageal motility disorder, esophageal webs and rings, and esophagitis [1].

**Table 1:** Significant lab values in a patient with diffuse esophageal spasm, Clostridium difficile colitis, and several other comorbidities

Test	Result	Reference range
WBC (white blood cells) x 10^9^/L	20,080	(4,000-11,000)
Neutrophils (%)	86.1	(50-70)
Hemoglobin (g/dL)	13.10	(12-16)
Hematocrit (%)	42.20	(37-47)
PLT (platelet count) x 10^3^/µL	264	(125-450)
Sodium (mEq/L)	141	(136-145)
Potassium (mEq/L)	2.9	(3.5-5.0)
Chloride (mEq/L)	116	(98-106)
Bicarbonate (mEq/L)	12	(23-28)
BUN (blood urea nitrogen, mg/dL)	38	(8-20)
Creatinine (mg/dL)	3.5	(0.50-1.10)
Anion gap (mEq/L)	13	(7-13)

**Figure 1:**
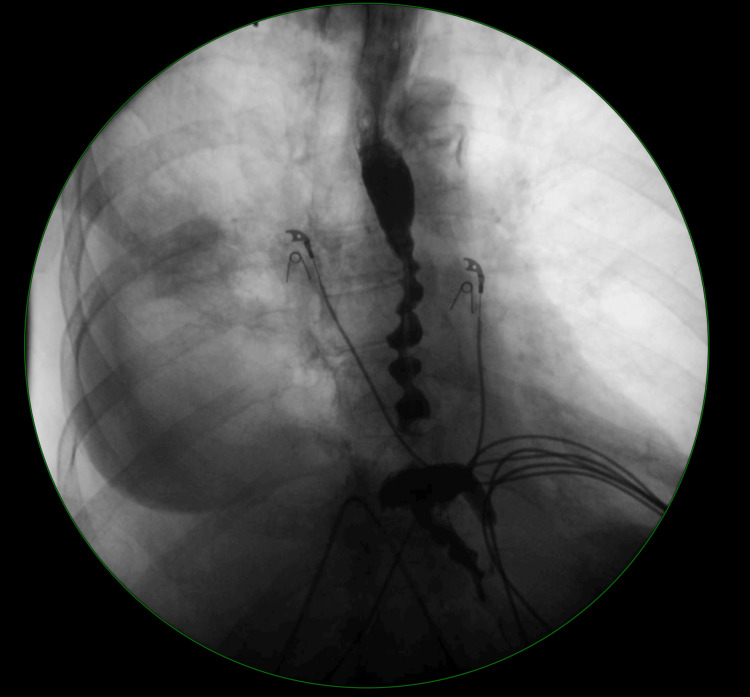
Barium swallow of a 91-year-old patient with diffuse esophageal spasm (DES) Notice the ‘corkscrew’ or ‘rosary-bead’ appearance to the esophagus.

The upper gastrointestinal series (barium swallow) revealed a lack of the primary stripping wave with to and fro motion within the esophagus with the corkscrew appearance of the esophagus as seen in Figure 1. Given her age, comorbidities, and current state, she was not a candidate for surgery. Pharmacologic treatment with metoclopramide before meals and nifedipine was initiated. The patient continued to progress to dysphagia with liquids and solids. The patient was then evaluated by surgery. During her initial evaluation with surgery, she reported swallowing issues over the last few years and increasing chest discomfort.

In light of the uniquely complex aspects of this case, gastroenterology was consulted. An upper gastrointestinal endoscopy with esophageal balloon dilation was performed. The patient’s gastroenterologist obtained informed consent for the procedure in paper form. Sublingual nitroglycerin was ordered post-procedure for pain secondary to esophageal spasm. In the following days, the patient's swallowing improved, and oral intake increased. It was recommended the patient follow up if problems recur or for repeat esophageal dilation. Management of the patient’s other comorbid conditions was continued for several more weeks. The patient was eventually released to home on hospice.

Consent for the publication of this report, to include images and details of the case was obtained from the patient and her daughter, who is her power of attorney. Consent for the performed procedure was obtained by the patient’s gastroenterologist in paper form.

## Discussion

DES is a rare esophageal motility disorder without a well-established treatment algorithm for evidence-based modalities. There are commonly delays in diagnosis, missed diagnoses, and often unpredictable outcomes for patients with DES [1]. In patients who are not candidates for the more invasive treatment options or are intolerant to medications, endoscopic pneumatic dilation can be an effective management strategy to improve outcomes for these patients [11].

Esophageal manometry is considered the gold standard for the diagnosis of DES [2]. Barium swallow has a higher sensitivity than endoscopy for detecting strictures but a lower overall accuracy for diagnosing achalasia [4]. Taking into consideration this patient’s age, comorbidities, long-standing history of GERD, and dysphagia, it was noted that strictures were likely. This led to the decision to use barium swallow to aid in diagnosis. The diagnostic criteria for DES have evolved in recent years after the introduction of high-resolution esophageal pressure topography [2]. The current diagnostic criteria include at least two premature contractions with a distal latency under 4.5 seconds with the assumption of normal esophagogastric junction relaxation [2]. There are no specified guidelines for the treatment of DES when first-line therapy is unsuccessful or there are contraindications to medication use. There are limited studies noting the effectiveness and long-term outcomes of the more invasive approaches like traditional surgical approaches, such as Heller myotomy or the relatively newer surgical approach POEM, in individuals with DES specifically [2]. Endoscopic Botulinum toxin injections are a more recently established approach. As of now, there is not yet enough data to support this as a primary treatment modality [5].

Endoscopic esophageal dilation is a traditional approach that can be overlooked by the vast array of possible treatment options. In subsets of patients, such as the elderly or immunocompromised, there is a need for less invasive treatment modalities that provide a solution longer than the duration of action of an oral medication. In this case, the patient was elderly and currently being treated for several other medical issues with multiple medications. Adding additional medications would increase the risk of drug interactions, side effects, and toxicity while only providing short-term relief. After a failed trial with metoclopramide and nifedipine, when weighing the risks and benefits of the various treatment options, it was decided to utilize a second-line treatment option, esophageal pneumatic dilation. This case is unique in demonstrating the successful management of a rare esophageal motility disorder in a high-risk patient using a more traditional, less invasive treatment approach. A previous study analyzing the long-term outcomes of esophageal dilation in 51 patients with diffuse esophageal spasm revealed that four years after esophageal dilation, 56.25% reported improvement in their symptoms [12]. There were no complications such as esophageal perforation [12]. This study concluded that based on their results, esophageal dilation in patients with DES is both safe and has beneficial long-term effects [12]. The study also noted there was no association found between size or type of dilator and outcome [12]. In comparison to this case, the previous study had a mean diagnosis age of 68.5 years and our patient had an age at diagnosis of 91 [12]. Additionally, our patient was being treated for multiple other comorbidities at the time of esophageal dilation. The patient discussed in this report was also released on hospice, which limits the ability for long-term follow-up. Although similar to the aforementioned study, the patient did report improvement in swallowing following esophageal dilation and was able to begin eating more allowing for the resolution of severe malnutrition. 

Recommendations for the management of DES include following evidence-based guidelines that are available for both diagnosis and treatment. In patients with other medical issues or contraindications to modalities listed in the utilized guidelines, clinical reasoning, and a multidisciplinary, approach including a thorough risks and benefits discussion and shared decision-making process with the patient should be used. The most up-to-date evidence-based recommendations for diagnosis include high-resolution esophageal manometry as the gold standard approach [3]. Criteria for diagnosis include at least two premature contractions with a distal latency of less than 4.5 seconds in at least 20% of wet swallows with the assumption of normal lower esophageal sphincter tone [3]. Barium swallow and endoscopy are considered ancillary tests but can provide additional information allowing differentiation of DES from other esophageal disorders [3]. As in this case, the demonstration of classic imaging and clinical presentation along with a thorough history may be sufficient in making the diagnosis of DES. Classic imaging reveals a ‘corkscrew’ appearance of the esophagus, as seen in this patient in Figure 1.

Limitations

This case report may be limited by generalizing the validity of the study and the inability to establish a causal relationship. The study may also be limited by the effects of the patient’s age, comorbidities, and their respective medical management.

## Conclusions

This report presented a patient diagnosed with DES after the incidental finding of classic imaging of an esophagus resembling a corkscrew on a barium swallow, all of which were complicated by the patient’s various comorbidities. This case adds to the literature by presenting a case of successful management of DES with esophageal dilation alone. More studies are needed to evaluate the treatment of DES in cases complicated by the presence of other serious medical issues. Further research is needed to gain support for all of the different treatments of DES. Research with special attention to complicating factors, such as advanced age, and commonly associated conditions, such as GERD and a history of smoking or alcohol use, need to be considered when studying the different approaches to management. In addition, more research on different approaches to diagnosis is needed to provide support for alternative approaches to diagnosis in certain cases.

## References

[1] Goel S, Nookala V (2020). Diffuse Esophageal Spasm. https://www.ncbi.nlm.nih.gov/books/NBK541106/#:~:text=Diffuse%20esophageal%20spasm%20is%20an,is%20associated%20with%20high%20morbidity..

[2] Salvador R, Costantini M, Rizzetto C, Zaninotto G (2012). Diffuse esophageal spasm: the surgical approach. Dis Esophagus.

[3] Lee EM, Park MI, Moon W, Kim KM, Park SJ, Kim HH (2010). A case of symptomatic diffuse esophageal spasm during multiple rapid swallowing test on high-resolution manometry. J Neurogastroenterol Motil.

[4] Goyal A, Chatterjee K, Yadlapati S, Singh S (2017). Health-care utilization and complications of endoscopic esophageal dilation in a national population. Clin Endosc.

[5] Patel DA, Yadlapati R, Vaezi MF (2022). Esophageal motility disorders: current approach to diagnostics and therapeutics. Gastroenterology.

[6] Sterling JL, Schey R, Malik Z (2018). The role of botulinum toxin injections for esophageal motility disorders. Curr Treat Options Gastroenterol.

[7] Vanuytsel T, Bisschops R, Farré R (2013). Botulinum toxin reduces dysphagia in patients with nonachalasia primary esophageal motility disorders. Clin Gastroenterol Hepatol.

[8] Okuda T, Higashino M, Osugi H, Maekawa N, Tanimura S, Kinoshita H, Wakasa K (1993). Case of diffuse esophageal spasm treated by long myotomy [Article in Japanese]. Nihon Geka Gakkai Zasshi.

[9] Kuwano H, Miyazaki T, Masuda N, Kato H, Kusano M (2004). Long myotomy of the esophagus and gastric cardia with a complete fundic patch procedure for diffuse esophageal spasm. Hepatogastroenterology.

[10] Kim JY, Min YW (2020). Peroral endoscopic myotomy (poem) for treating esophageal motility disorders. Clin Endosc.

[11] Pehlivanov N, Pasricha PJ (2006). Medical and endoscopic management of achalasia. Nature News.

[12] Almansa C, Eslick GD, DeVault KR, Achem AR (2009). W1870 long outcome of esophageal dilation in patients with diffuse esophageal spasm. Gastroenterology.

